# Choking at Night: A Case of Opercular Nocturnal Frontal Lobe Epilepsy

**DOI:** 10.1155/2013/606385

**Published:** 2013-12-05

**Authors:** Geetanjali Rathore, Paul Larsen, Manish Parakh, Cristina Fernandez

**Affiliations:** ^1^Baylor College of Medicine, Pediatric Neurology, 6701 Fannin street, Houston, TX 77033, USA; ^2^University of Nebraska Medical Center, Pediatrics and Neurology, Children's Hospital and Medical Center, 8200 Dodge Street, Omaha, NE 68114, USA; ^3^Umaid Women and Children's Hospital, Pediatric Neurology, Sivanchi Gate, Geeta Bhawan Road, Pratap Nagar, Jodhpur, Rajasthan 342002, India; ^4^Children's Physicians, Pediatrics, 601 North 30th Street, No. 6820, Omaha, NE 68131, USA

## Abstract

Frontal lobe seizures have a tendency to occur in sleep and in most cases occur exclusively in sleep; these individuals are said to have nocturnal frontal lobe (NFLE). NFLE can be difficult to distinguish clinically from various other sleep disorders, particularly parasomnias, which also present with paroxysmal motor activity in sleep. Interictal and ictal EEG findings are frequently unremarkable or nonspecific in both parasomnias and NFLE making the diagnosis even more difficult. Nocturnal epilepsy should be suspected in patients with paroxysmal events at night characterized by high frequency, repetition, extrapyramidal features, and marked stereotypy of attacks. Here we present a 13-year-old female who was extensively worked up for choking episodes at night. On repeat video EEG she was found to have frontal opercular seizures. Once on Carbamazepine, her seizures completely resolved.

## 1. Introduction

Nocturnal paroxysmal events are commonly misdiagnosed and underdiagnosed. Differentiating epileptic seizures from sleep disorders can be difficult based on history alone. Absence of epileptiform activity on a scalp EEG interictally and ictally makes the diagnosis a further challenge. Nocturnal chocking may be a predominant symptom of both nonepileptic sleep disorders and epileptic seizures. Nocturnal epilepsy should be suspected in patients with paroxysmal events at night characterized by high frequency, repetition, extrapyramidal features, and marked stereotypy of attacks.

## 2. Case Report

The patient is a 13-year-old female seen in clinic for evaluation of nocturnal spells of unknown etiology. Patient was doing entirely well until 8 months prior to presentation. Since then, the patient would wake up in the middle of the night suddenly gasping for air, in severe distress due to shortness of breath. During these episodes she experienced severe nausea, hyperactive bowel sound, retching, and choking sensation. These episodes were followed by hypersalivation, bitter taste, and tongue numbness lasting for about 10–15 minutes. These episodes lasted for about 20–40 seconds and occurred daily about 4-5 times a night. During these episodes the patient was aware of her surroundings, followed commands, but was unable to communicate back verbally. She showed no signs of incontinence, rhythmic shaking movements, or tongue biting. She denies any aura and these episodes occurred only at night. Patient was born via C-section secondary to nuchal cord. The pregnancy, delivery, and postnatal course were unremarkable. Past medical history is unremarkable and there is no history of trauma, febrile seizure, or developmental delay in the patient or her family.

Physical examination is normal including her memory, speech, cranial nerves, coordination, and motor and sensory examination. The patient had been evaluated by neurology, ENT, pulmonology, and GI. She had an extensive workup including complete blood count, serum chemistries with liver function, MRI of brain, 3 EEGs, sleep study, video EEG, pH probe, and upper GI endoscopy. All these studies were normal, expect that the gastric biopsy showed *H. pylori* for which she was treated with 14 days of antibiotics. Patient was started on omeprazole, with questionable benefit. Patient was also started on a trial of valproate which was titrated up to 750 mg daily. It was discontinued as it did not help.

We repeated a video EEG and during this study the patient had 9 events. The ictal EEG showed low voltage, fast activity, with definite changes in background. The interictal EEG showed right frontocentral sharp waves. After these findings an MRI on 3T magnet with special focus on frontal and temporal lobe was performed. It showed scattered nonspecific tiny white matter hyperintensities on the T2 coronal thin cuts. The largest 5 mm lesion was seen in the right frontal lobe. The patient was initially started on levetiracetam, but the seizures were not controlled, so she was switched to Carbamazepine. On follow continued follow up the patient has remained seizure free for over 2 years now.

## 3. Discussion

The diagnosis of paroxysmal events in sleep represents a significant challenge for clinicians in differentiating epileptic seizures from nonepileptic sleep disorders. Nocturnal seizures, in particular, frontal lobe seizures, are more difficult to identify as they display a wide range of bizarre clinical features combined with often normal or nonspecific interictal and ictal EEG [1]. Nocturnal frontal lobe epilepsy (NFLE) is an epileptic seizure disorder consisting of a heterogeneous group of paroxysmal sleep-related disturbances [2]. In a major study done by Provini et al. about 97% of these episodes occur during the non rapid eye movement (NREM) stage of sleep, 69% of which occur during light sleep (stages 1 and 2). Only 3% occurred during REM sleep. The age of onset varies widely but is seen more often from infancy to adolescence, with 14 years being the mean age of onset. NFLE tends to persist in adulthood. In contrast parasomnias usually present in early childhood and generally resolve by adulthood. It has a higher incidence in males. A familial clustering of NFLE has been described [3, 4]. Scheffer et al. [5] introduced the term autosomal dominant NFLE (ADNFLE) showing autosomal dominant transmission of NFLE in 5 families. Philips et al. linked chromosome 20q13.2 with the inheritance of ADNFLE. New genes are being identified, like mutation of the M2 domain of CHRNA4 gene which was identified by Steinlein et al. [6] in a Norwegian family. Patients with NFLE have a strong family history of parasomnias.

In NFLE the seizures tend to cluster, with multiple episodes occurring within a couple of hours. On an average 3–8 events occur per night [2]. Some patients having greater than 20 episodes per night have been reported [7]. Parasomnias characteristically occur quite infrequently, on an average 1–3 times per month [8]. NFLE seizures are usually brief, with the majority averaging less than 2 minutes. Of the 660 seizures recorded by Provini, the longest lasted 3 minutes. Parasomnias are usually described as relatively prolonged events, usually >5 minutes in duration, although they may be briefer [9]. Frontal lobe seizures may occur anytime during sleep but most commonly occur within 30 minutes of sleep onset [7]. This correlates with the neurophysiologic findings of frontal lobe epilepsy, which demonstrate that these seizures most commonly occur during stage 2 of NREM sleep [10]. K complexes may represent an important factor in the onset of NFLE seizures as they tend to cluster with quasiperiodic repetition at a rate similar to that of K complexes [11]. Polygraphic recordings done by Provini et al. showed that K complexes often coincided with or even preceded the ictal EEG and autonomic manifestations. In contrast disorders of arousal occur during deep NREM sleep (stages 3 and 4) thus usually occurring 90 minutes to 2 hours after sleep onset [12, 13].

During parasomnias, patient is unaware of surroundings and usually has no recollection. In NFLE patient is aware of surroundings and usually has a clear recollection of most of these events [1]. The interictal EEG is often normal and even the ictal EEG may be equivocal, making the diagnosis of nocturnal seizures even more confusing. As these seizures may arise from deep frontal regions, routine scalp EEG may be inadequate to detect clear-cut epileptic abnormalities, even during the episode. In a large scale study done by Provini et al. 44% ictal and 55% interictal EEG were uninformative, either being normal or masked by muscular artifacts. Sphenoidal EEG electrodes showed ictal epileptic activity in 13 patients with normal scalp EEG.

Based on their semiology and duration, NFLE has been distinguished into 3 major categories. (1) Paroxysmal arousals are characterized by abrupt and brief episodes of stereotypical motor activity. (2) Nocturnal paroxysmal dystonia seizures are characterized by complex dyskinetic and dystonic motor attacks lasting <2 minutes. (3) Episodic nocturnal wandering consists of stereotyped agitated somnambulism [2]. Frontal lobe seizures may arise from any of the frontal lobe areas, including orbitofrontal, frontopolar, dorsolateral, opercular, supplementary motor area, motor cortex, or cingulate gyrus. Details obtained about the seizure semiology may help to identify the specific frontal region of onset.

Our patient presented with seizure activity in the frontal opercular region. The cerebral operculum refers to the portion of the frontal, parietal, and temporal lobe adjacent to the Sylvian fissure and overlying the insula. The anterosuperior part of the operculum formed by the inferior frontal gyrus contains the frontal operculum. Opercular seizures usually manifest with oral symptoms like mastication, swallowing, salivation, and gustatory hallucinations. Epigastric aura can be seen as well as fear and autonomic changes like tachycardia [14]. Many patients specially with ADFLE have an aura which wakens them most commonly with a sensation of choking or dyspnea [5].

Cognitive and behavioral disturbances, commonly encountered in other frontal lobe epilepsies, have not been reported in patients with NFLE. NFLE usually does not show a tendency to spontaneous remission. Carbamazepine completely abolished seizures in 20% of the treated patients and reduced the seizures by about 50% in another 48% of the patients. About a third of patients have seizures resistant to antiepileptic drug treatment. Patients with drug resistant NFLE are usually good candidates for surgical treatment. Seizure freedom rates of as high as 75% have been reported [15]. Frontal lobectomy showed a 55.7% seizure freedom rate in a study done by Jeha et al. [16] on 70 patients. NFLE is not a rare variant of epilepsy. It accounted for about 13% of polysomnography studies done on patients with nocturnal paroxysmal disorders over the past 20 years [2]. NFLE is generally a benign epilepsy [17]. Some degree of benignity is due to the fact that these seizures mostly occur at night. Some patients do not feel incapacitated by them and choose not to get treated. However, this is not the case in all patients; persistence of nocturnal seizures may have a significant negative impact on the quality of life of some patients. They may experience excessive daytime somnolence.
